# Structural constraints on the evolution of the collagen fibril: convergence on a 1014-residue COL domain

**DOI:** 10.1098/rsob.140220

**Published:** 2015-05-20

**Authors:** David Anthony Slatter, Richard William Farndale

**Affiliations:** 1 School of Medicine, University of Cardiff, Tenovus Building, Cardiff CF14 4XN, UK; 2 Department of Biochemistry, Downing Site, University of Cambridge, Cambridge CB2 1QW, UK

**Keywords:** collagen, exon structure, D-period, cross-links

## Abstract

Type I collagen is the fundamental component of the extracellular matrix. Its α1 gene is the direct descendant of ancestral fibrillar collagen and contains 57 exons encoding the rod-like triple-helical COL domain. We trace the evolution of the COL domain from a primordial collagen 18 residues in length to its present 1014 residues, the limit of its possible length. In order to maintain and improve the essential structural features of collagen during evolution, exons can be added or extended only in permitted, non-random increments that preserve the position of spatially sensitive cross-linkage sites. Such sites cannot be maintained unless the twist of the triple helix is close to 30 amino acids per turn. Inspection of the gene structure of other long structural proteins, fibronectin and titin, suggests that their evolution might have been subject to similar constraints.

## 2. Introduction

Mammalian collagens are the most abundant extracellular proteins, providing the framework upon which the extracellular matrix is assembled and within which cells are organized to form tissues and organs. They are characterized by repeating G-X-X' triplets, where G is glycine, X is commonly proline (P) and X’ is commonly hydroxyproline (O). Three G-X-X'-containing polypeptide strands assemble to form the triple-helical COL domain, which, with short non-helical telopeptide extensions, is known as the tropocollagen molecule. In the modern fibrillar collagens, many such helices assemble to form a fibre, where each triple-helical monomer is offset from its immediate neighbours by integral numbers of D-periods (234 residues in modern collagens), and the length of the helix corresponds to about 4.3 D-periods. A gap of about 0.7 D-periods exists between coaxial helices within a fibre. Fibres are stabilized by covalent cross-links between specific sites in adjacent helices, described in more detail below.

Here, our objective is to construct an evolutionary sequence explaining the current state of modern collagens from their most basic starting point, without requiring overtly improbable events. Our underlying assumption is that once a fibre-forming collagen helix of any length is in place, further development must preserve both its triple-helical form and the axial orientation of the helix within the supramolecular structure of the fibre if the stabilizing cross-linking is to be preserved. We also assume that there is a selective advantage to this lengthening, in terms of greater stability, accommodating extra binding sites and better scaffold properties.

**Figure 1.**
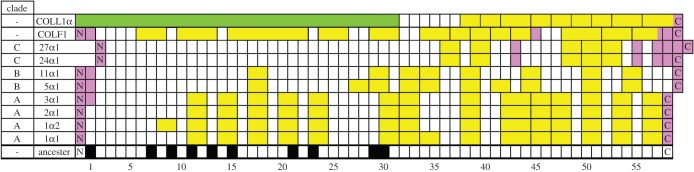
Gene structure of vertebrate and freshwater sponge fibrillar collagens. The 57-exon ancestral fibrillar collagen helix had either 54-base pair (white squares) or 45-base pair (black squares) exons, with additional N- and C-terminal exons. This diversified into invertebrate collagens (COLL1α, COLF1), and within vertebrates, A–C clade collagens. Many exons have merged (yellow, green) and a few have changed in length (pink) since the formation of the ancestral collagen.

Such work must commence with investigation of the known fibrillar collagens: ColF1 from freshwater sponge [1,2]; Coll1α from sea urchin [3]; A-clade collagens Cols Iα1, Iα2 IIα1, IIIα1; B-clade collagens Vα1, XIα1 and C-clade fibril diameter regulators XXIVα1 and XXVIIα1 [4], all from fish, mammals and others [5], suggested a common 57-exon ancestral COL domain (figure 1). Its exons were of either 45 bp (black squares) or 54 bp (white squares). In all modern collagens, some exons have subsequently fused together (yellow, green). Invertebrate and the more recently described C-clade collagens have deviated further from this ideal with significantly more exons of non-standard length (pink) [1–7]. Outside the main helix featured in figure 1, only the heavily conserved C-terminal propeptide (NC1) domain required for aligned helical folding is conserved across fibrillar collagens, where invertebrate collagens are distinguished from the vertebrate A–C clade collagens by a seven-residue deletion [6]. From all these data, it has been inferred that the first exon of the collagen triple helix contained a stretch of 54 bases encoding (GPO)_6_ [8]. In addition, there are exons at the N- and C-termini of the helix that encode one or five G-X-X' triplets and the adjacent non-helical telopeptides, respectively [9].

Our proposal describes events that pre-date the formation of the common collagen ancestor, thus pre-dating formation of the metazoan kingdom, as there are fibrillar collagens from the same ancestor seen in choanoflagellates [6]. Like most proteins, the collagen triple helix has limited thermal stability, where substitution of the X and X' amino acids within the thermally optimal (GPO)*_n_* sequence reduces the temperature at which the helix unwinds. Accordingly, (GPO)_6_ represents the shortest collagen that can assemble as a helix in cold water [10], the environment where collagen must have evolved [11,12]. Short GPO polymers have limited biological utility, although a (GPO)_10_ peptide might possibly serve as an extracellular matrix scaffold by virtue of forming fibril-like aggregates at neutral pH and high concentration [13]. The absence of short collagen helices from current biological systems suggests that the modern, longer, proteins with greater diversity of primary sequence offer greater benefit. In constructing the evolutionary lengthening of fibrillar collagen below, we use only the clues granted by the exon lengths themselves along with the general helical form of their encoded peptides. This is because protein, DNA and exon sequence analysis of collagen type Iα1 genes did not yield any clues that informed on which older exons may have been duplicated back into the gene in order to lengthen the protein (see the electronic supplementary material), probably due to mutation and diversification of collagen Iα1 sequences since the formation of the ancestral collagen over 540 Ma. This futile analysis was in contrast to analyses between various whole collagen sequences and exon structures, from which important conclusions have been made [11,14].

## 3. Theorem

The extension of the primordial collagen gene must have occurred before any deviation from the perfect (GPO)_6_ sequence, to maintain thermal stability. One possibility is that 45 bp and 63 bp exons, the latter seen in the non-fibrillar collagens VI, VII and XIX, can evolve from a 54 bp exon by unequal recombination [11,14]. The chance removal of the intervening nucleotides encoding the flanking residues is rewarded by a longer, more stable helical structure, such as the protein **(X)_n_(GPO)_6_**(GPO)_5_(X)_c_, where bold or normal type denotes sequence coded by the two exons, and **(X)_n_**/(X)_c_ represent primitive non-helical telopeptides. Another possibility is that an initial 54 bp exon could be copied back into itself, forming the three-exon sequence (X)_n_GPO**(X)_n_(GPO)_6_(X)_c_**(GPO)_5_(X)_c_, where removal of internal X sequences improves helix stability as before.

**Figure 2.**
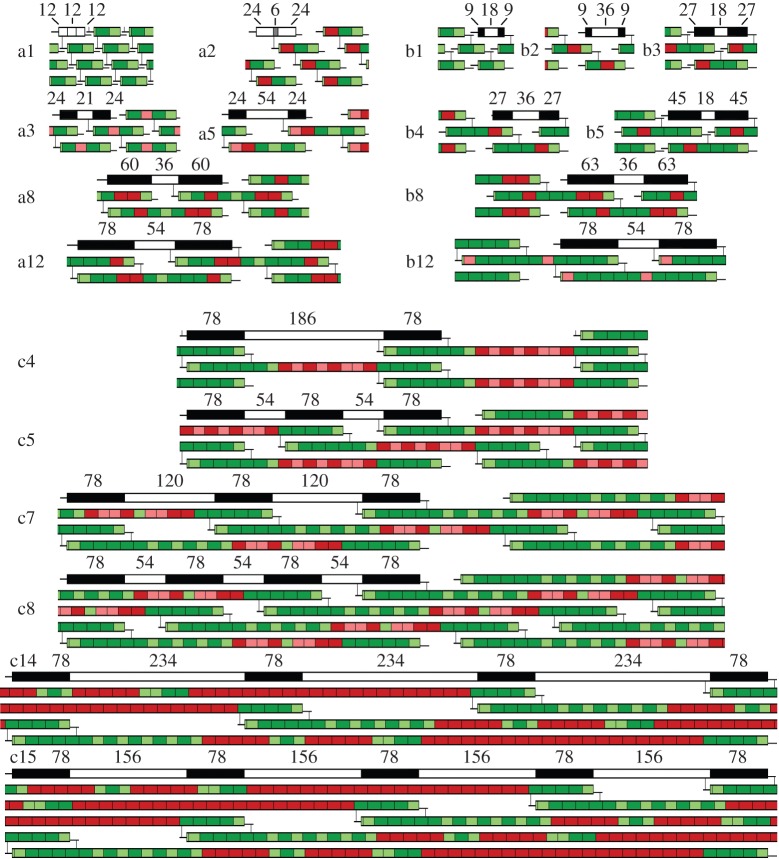
Evolution of the collagen molecule in incremental steps a1–12 or b1–12 followed by c1–15. In each fibril (a1, a2, a3, b8, c8, etc.), the helical overlap (black) and gap (white), and for a2 double-gap (grey), regions are shown on one molecule. Dark green/red rectangles are encoded by 54 bp exons, whilst light green/pink rectangles are encoded by 45 bp exons (or are the N/C termini). Red/pink segments correspond to those exons added most recently. The electronic supplementary material, table S1 shows all steps numerically.

This three-exon example, (X)_n_GPO**(GPO)_6_**(GPO)_5_(X)_c_, allows evolution of collagen to proceed in earnest, as shown in figure 2, a1/b1. There, 54 bp exons are shown in dark green, 45 bp (and 9 bp) exons in light green, and they are placed together to show the entire collagen helix as a bar with linear telopeptides on its end.

Cross-links between lysine and hydroxylysine residues in the helix and telopeptides of adjacent tropocollagen molecules are required for the formation of stable, organized fibrils. These require point mutation in a central exon X or X' proline codon to yield lysine, and the oxidation of a telopeptide lysine to (hydroxy)lysine aldehyde (figure 2, step a1/b1). When in proximity, these react to form a Schiff's base between helices, shown by lines joining the helices to telopeptides (figure 2). Formation of more permanent covalent links [15–19] happens later over time. Regardless of this cross-link location, collagen extension by exon addition can occur: the schemes a and b in figure 2 are shown as two possible examples, assuming N-telopeptide and C-telopeptide cross-linked collagens, respectively. Exon additions shown in red (54 bp exons) or pink (45 bp exon) could have been effected by unequal recombination, transposition or saltatory replication. As the collagen lengthens, the gap region (white bar) will be kept to a minimum as denser fibres allow more non-covalent interactions between the helices [13].

Modern fibrillar collagens form cross-links at each end, increasing stability, fixing the axial alignment of successive triple helices and defining the longitudinal, one D-period displacement between adjacent helices. For type I collagen, cross-links are between helix residue 87 of its 1014 and the C-telopeptide of another tropocollagen molecule, and between helix residue 930 and another N-telopeptide. Transmission electron microscopy reveals alternating striations, with light ‘gap’ regions and dark ‘overlap’ regions along the fibril, as shown schematically for one helix within each panel of figure 2. A gap and overlap together are called a D-period, and fibrillar collagens are typically four D-periods of 234 amino acids (residues), one overlap region of 78 residues, with telopeptides of 10–20 residues. For every five adjacent collagen molecules in the overlap region, one ends at the gap and only four traverse the gap (figure 2 step c15). Therefore, the spacing of a collagen molecule within a fibril is five D-periods, prompting one to ask the question: why five?

**Figure 3.**
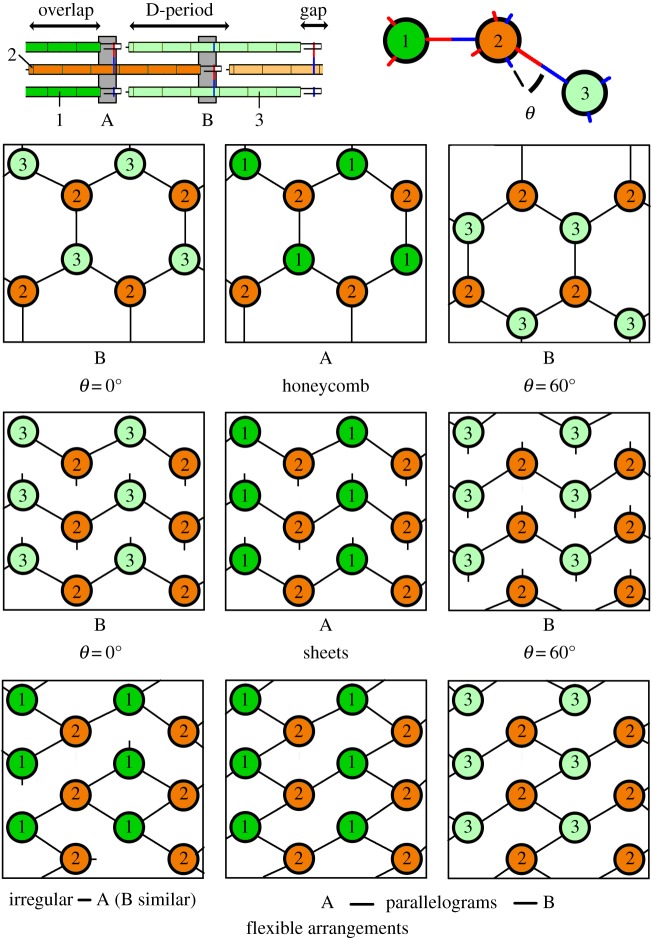
Possible cross-sections of early collagen fibrils with two D-periods. A sideways view is shown top left of a simple 2 D-period fibre. Each of the nine diagrammed squares shows possible cross-sectional topologies of helices 1–3 at cross-sections A and B. Possible rigid arrangements such as a honeycomb or sheets will have subtly different topologies at B depending on the angle *θ* (top right). If the cross-linking is more flexible, irregular or parallelogram-type arrangements are possible. See the electronic supplementary material for additional comments.

With just one set of cross-links, lengthening of collagen was straightforward. Telopeptide flexibility can accommodate axial rotation between the helix and telopeptide cross-link sites. However, two constraints still apply: first, exons encoding helix between the helix and telopeptide cross-link sites must first increase the size of the gap region (e.g. figure 2, b1–b2). Exons added elsewhere can then increase the size of the overlap region and decrease the size of the gap region (e.g. figure 2, b2–b3), causing different packing arrangements. Second, the cross-sectional packing of the fibre places constraints on collagen extension even with one collagen cross-link per helix. A seven-exon collagen is shown in figure 3 (top left). Looking down through the cross-sections A and B from figure 3, top left, the red C-terminal telopeptide (hydroxy)lysine aldehyde residues of helix 1 point out radially, linking to the blue N-terminal cross-linking helical (hydroxy)lysine of helix 2 (figure 3, top right). Depending on the helical twist, the angle *θ* between this and the red C-terminal telopeptide link of helix 2 to the blue N-terminal link of helix 3 may vary between 0° and 120°, where angles of 60°–120° result in topologically similar reflections of angles of 60°–0°. Four of the nine square panels below now display cross-section A (centre and bottom left), each circle representing a helix, while the remaining panels display topologies at cross-section B, depending on whether *θ* is 0°/120° or 60°. Honeycomb structures could form (top row), but quasi-hexagonal sheet-like arrays, observed in modern collagen fibres [20], have more extensive hydrophobic contacts (middle row). These latter arrays only support two cross-links per collagen molecule, but also allow one chain to be replaced with a non-cross-linking chain such as collagen type Iα2. Other topologies might occur if cross-links are flexible, forming randomly, including parallelogram-type cross-linking or perhaps entirely irregular cross-linking patterns (bottom row); however, no such cross-linking pattern has been observed.

When the helical overlap of triple helices reached 78 residues (figure 2, a12, b12), a second set of cross-links formed, probably required to strengthen longer fibrils. This defined the 87-residue distance between the helix N-terminus and the first cross-linking lysine, and the 85 residues between the second cross-linking lysine and the helix C-terminus. If either changed at a later date, the cross-linking residues would misalign, with lethal effect. This cross-link could have occurred earlier or later, resulting in the evolution of different collagens (electronic supplementary material, table S2), but an overlap/gap size of 78/54 at this point is mathematically versatile as the number of D-periods increases.

**Figure 4.**
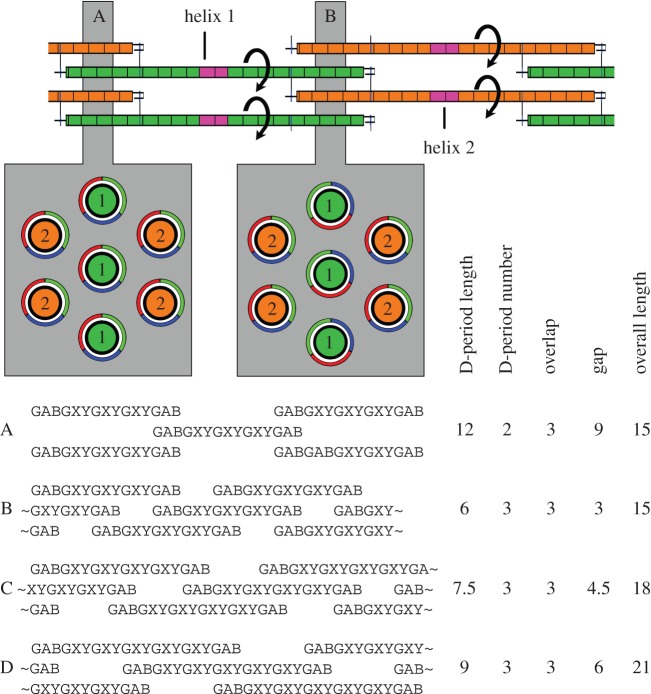
Topological considerations upon collagen extension. Top: If introduction of new sequence into the helix (pink) rotates the helix by an integral number of turns, the packing of the helices at B is identical to the cross-sectional packing at A (grey box A). If the helix C-terminus, however, is rotated by a non-integral number of turns, the packing changes (grey box B). Bottom: Schematic to show that there are restrictions on collagen upon rearranging to include more D-periods. Short ‘collagens’ are shown for clarity (see text). A two D-period collagen with 12 residues in its full D-period (*a*) can rearrange to a collagen with a six-residue D-period (*b*), but a two D-period collagen with 15 residues cannot do so without misalignment (*c*). Add another three residues, and the homogeneity of the collagen is restored with a nine-residue D-period (*d*). See the electronic supplementary material for additional comments.

Exon addition must now encode sequence within the helix but between its cross-link sites, extending the gap region, which can only be reduced subsequently by rearrangement to give more D-periods. But each addition must encode integral numbers of helix turns. Figure 4 shows a collagen molecule with two new exons added (pink rectangles at top). If these introduce exactly one turn into the helix, then the interactions in the overlap region of cross-section B result in an orientation of glycine (green), X (blue) and X' (red) residues that is the same as cross-section A. A non-integral number of turns resulting in, say, a 120° anti-clockwise rotation may still allow cross-link formation, but the helix packing in cross-section B is now radically different. The initial distance between the cross-links is now set at 39 residues (figure 2, a12, b12). This could be approximately 1⅓ turns at 30 residues per turn if the helix has 10_3_ symmetry as initially proposed from X-ray diffraction of collagen fibrils [21] and more recently from some peptide structures with real collagen sequence [22,23], or it could be two turns at 21 residues per turn if the helix has 7_2_ symmetry as suggested (controversially) from more recent X-ray diffraction data [9,24] and observed in (GPP)_*n*_ or (GPO)_*n*_ peptide crystal structures [25]. The *θ* angle between the N- and C-terminal cross-links in the helix becomes defined: 120° for 10_3_ symmetry, or 0° for 7_2_ symmetry.

Evolutionary extension of a 7_2_ helix now seems unlikely. One exon of 45 or 54 bases codes for 15 or 18 residues, less than one turn, whereas two exons totalling 99 or 108 bases encode 33 or 36 residues, about 1.5 turns. To achieve a multiple of approximately 21 residues, at least 4 exons must be inserted within the body of the gene simultaneously, encoding e.g. 66 residues (45-54-45-54 bp exons), approximately 22 residues per turn. By contrast, in the looser 10_3_ helix, only two exons, totalling 99 bases, can code approximately 33 residues for a one-turn extension. The exon addition scheme shown in figure 2*c* therefore adds exons in pairs of 45–54 bp for steps c1–c4. Collagen genes with only 54 bp exons and not 45 bp exons would be selected against as they must extend the helix by 36 rather than 33 residues at a time, further from the 10_3_ ideal.

As exons are added, the gap region becomes so long that the fibril may become unstable, having fewer contacts between helices. Rectifying this, illustrated in figure 2, c4–5, a 342 residue helix can take either of two conformations, where the helix and a single gap total two or three D-periods. The three D-period conformation then increases contact, halving the D-period length to 132 residues, and cutting the gap to 54 residues. This does not affect the cross-links or the overlap interactions, where packing is tighter. While this reshuffle needs a gap region big enough for the telopeptides, there are other restrictions. Illustrating these, short, theoretical (GXY)*_n_* proto-collagens with cross-linking GAB triplets are shown in figure 4 (bottom) with just a single chain per helix for clarity. For the two D-period fibril A (akin to figure 2*c*, 4), the 12-residue D-period length is divisible by two, the existing number of D-periods, and this allows a rearrangement to three D-periods of six residues: fibril B. An 18 residue collagen with a D-period of 15 residues, not divisible by two, cannot rearrange without either having an irregular gap distance or having glycine out of phase: fibril C. Furthermore, to extend the three D-period fibril B, it must lengthen two triplets, a number divisible by (D-periods minus 1), to form a 21-residue collagen with three D-periods of nine residues: fibril D. The gap region lengthens to six residues. Lengthening fibril B by just one triplet yields the dysfunctional fibril C again.

Therefore, a three D-period collagen is forced to elongate by two full turns at a time, with the number of added residues divisible by 6 to keep cross-link and side-chain orientations correct. Again, while four exons totalling 198 bases (66 residues) is close to two turns of a 10_3_ helix, a 7_2_ helix can only be extended by adding at least four turns of helix in one block (84 residues), encoded by three 54 bp exons plus two, rarer, 45 bp exons. Adding extra D-periods above three also requires cross-links to be deployed in sheets akin to those shown in figure 3, invalidating the honeycomb structure.

After addition of two identical batches of four exons (10_3_ helix) in this manner (figure 2, c7), the resulting three D-period, 474-residue collagen can reorganize to contain four D-periods (figure 2, c8). This time, the D-period length reduces by 1/3 from 198 to 132 residues, and the gap is again reset to 54 residues. Extension of the 10_3_ helix can occur again in steps (figure 2, step c9–c14), exactly three turns at a time with 90 residues from five 54 bp exons. The collagen extends to 1014 resides, rearranges from four to five D-periods (figure 2, c14–15), reducing the D-period by 1/4 from 312 to 234, finalizing the ancestral gene. As a modern collagen fibril formed from monomeric collagen *in vitro* thickens by a defined number of helices per D-period [26], this D-period lengthening supports more helix–helix interaction and greater tensile strength, allowing the collagen fibril to become narrower.

Upon forming the 5th D-period, one might suggest that collagen would lengthen four turns at a time, adding something like five 54 bp and four 45 bp exons encoding 120 residues, resulting in a 264 residue D-period length. However, the resulting number series from sequential extensions (234 + 30*n*) never yields a number divisible by five, required for any rearrangement from five to six D-periods. As residues must be added in groups divisible by four (D-periods minus 1), the nearest allowable additions to 120 residues are 108 or 132 residues, but this adds helix with a pitch of 26 or 34 residues, far from the canonical 10_3_ helix. Therefore, a collagen with six D-periods is much harder to attain. For instance, adding 13 exons in one batch could insert a whole D-period of 234 residues, eight turns of the helix, but then the collagen must instantly realign to six D-periods, as the number of added residues is not divisible by four. This also involves the duplication and re-insertion of a large (approx. 3500 bp) stretch of DNA. Any further extension of the protein over 1014 residues then has dubious value, as it can only extend the gap region, reduce inter-helix contact and destabilize the fibre. Whatever the reason for settling at 1014 residues, it is only at this point that an extra D-period requires the addition of a large group of exons. Moreover, there is no fibrillar structure that allows either five or six D-periods to coexist in the same collagen molecule. On the other hand, the evolution of collagen as described can explain the presence of a block of 23 contiguous 54-base pair exons, which only has a 1.3% chance of occurring if the 45 and 54 bp exons between the two cross-linking sites were integrated without restriction.

## 4. Discussion

We have demonstrated a path retracing the early evolution of collagen in a logical manner that respects the requirement for the correct orientation of cross-linking lysine and hydroxylysine residues. This path cannot predict when the ancestral NC1 domain was added to the C-terminus to aid alignment and folding of the three helical chains, as the helix sequence itself may have been adequate for this purpose initially [27], but the NC1 domain certainly pre-dates any diversification of the ancestral collagen. Likewise, most of this evolution will have taken place before collagen GPO prototype sequence mutated to include a huge diversity of protein binding sites as new functions were acquired. There are a number of receptors on the platelet surface, for example, and plasma glycoproteins that bind collagen [28,29], but ancestral collagen evolved in an organism with no cardiovascular system. Therefore, early collagens must have been able to accept a much wider spectrum of mutation that allowed them to acquire these specific functions, to a point where modern day collagens leave no trace of which exons were copied from one another, and furthermore have fewer locations that can be mutated without consequences [30]. The present analysis differs from conventional works on exon structures [31–33], which typically construct phylogenetic trees based on when exons combine [32], or are shuffled, thereby categorizing proteins into clades. In those works, there is no attempt to look at the function of each individual exon or the evolutionary advantage gained from adding specific exons, where both are required for reverse engineering of ancestral genes.

Similar constraints upon exon addition may apply to the evolution of other long structural proteins in which the spatial relationship between domains needs to be maintained. The gene structures of titin and fibronectin (electronic supplementary material, tables S4 and S5) parallel that of collagen, suggesting that the process proposed here might apply to other large structural proteins, illuminating what could be perceived as an intractable evolutionary problem.

## Supplementary Material

Slatter & Farndale ESM.pdf
