# Supplementary material for: Structural constraints on the evolution of the collagen fibril: convergence on a 1014-residue COL domain
Source: Open Biol. 2015 May 20;5(5):140220. doi: 10.1098/rsob.140220 (PMC4450265; doi:10.1098/rsob.140220)
Supplement: Slatter & Farndale ESM.pdf [file rsob140220supp1.pdf]

Structural constraints on the evolution of the collagen fibril:
Convergence on a 1014-residue COL domain.

David A. Slatter[1], Richard W. Farndale[2]

[1]Tenovus Building, School of Medicine, University of Cardiff, CF14 4XN, UK
[2]University of Cambridge, Department of Biochemistry, Downing Site, Cambridge CB2 1QW, UK.

Supplementary text.

Alternative evolution routes that may have been taken by fibrillar collagens:- The data shown in figure 2 is presented in tabular format in suppl. table 1a-c. The sections (a-c) of the table correlate to the sections (a-c) in figure 2 and are described in the text. Distances from the lysines of the KGHR (cross-link) consensus sequences are measured inclusive of the lysine. As mentioned in the main text, there are some complexities in figure 2-a2. The negative overlap shown there (grey) and in suppl. table 1a ("-6") is describing a section of a 3 D-period fibril that would in effect be a double-gap (i.e gap 24, double gap 6), where only one in three collagen molecules have a helical section that straddles that cross-section of the fibril. There is also a less intuitive, more complex, and better packed 5D-period alternative to 2-a2 with gap of 9 residues, and a double gap of 6 residues. Either of those packing arrangements can rearrange to 2 D-periods on addition of another exon. Later steps may require a second cross-linking (CL) or another re-arrangement of the number of D-periods (R).

In the main text, it is commented that if the second set of cross-links occurs at a different time, different final collagens can form.

(a) Suppl. table 2a gives an example of one of these collagens, which could have been longer than the modern 1014 residue fibrillar collagens, suggesting that type I collagen may represent an evolutionary cul-de-sac. However, longer collagens have more D-periods, much reducing the cross-link density, which in itself may have consequences.

(b) Suppl. table 3a-c, shows a possible evolutionary route for collagens using 63bp exons, where exons of that size would steer evolution to favour a collagen with 7/2 helix symmetry in the same manner that exons of 45 and 54bp favoured a 10/3 symmetry. Exons of 63 bp are found in non-fibrillar collagens, and would code for exactly one turn of a 7_2, helix, circumventing many complexities with respect to collagen helix extension. If native collagen really is a 7_2 helix, it therefore seems odd for it to have evolved from a collagen using 54bp and 45bp exons in favour of 63bp exons given that a perfectly viable scheme for a 63bp exon collagen development can be constructed.

In each case, the location of the cross-linking sites within the original 2 or 3 exons modulates the intial growth of the collagen. For **suppl. tables 3a-c,** the cross-link site is the 12th amino acid residue of the 63-base exon, allowing a similar structure at steps a12 or b12, and the copying of the helical cross-linking exon to form the second cross-linking exon. The mutation to the cross-linking lysine could be elsewhere. All this would mean is that a second mutation is required for the second cross-link site, or that spacing between the telopeptide cross-links and the end of their own helices would need to differ from that seen in modern collagen I: both are reasonable possibilities. For **suppl. tables 1-2,** we used the locations of the actual cross-link sites as a guide, restricting the possibilities to what we show.

There is some room for the helix content of the telopeptide exons to have varied over time, but this will not be possible once both sets of cross-links have been established as it will cause the links to misalign. It may even cause problems the instant any cross-link is inserted, allowing no room for change.

Examination of DNA and translated sequences:- We looked for evidence that some exons may be more closely related to each other than others. Exons 31-37 as a group were similar to exons 44-50 ($P<0.01$), and thus a group of 7 exons from one of 44-50 or 31-37 could have been copied into the other during the final stages of gene evolution. These exons are however exactly one D-period apart in the sequence, so it is possible that these similarities are due to convergent evolution once the COL domain had all its exons. Others such periodicities in the collagen sequence have been spotted, which presumably evolved since the exon framework was laid down (1). Further attempts to find which pieces of gene had been copied and reinserted were futile. Only the exon structure, the core Gly-X-X' sequence motif, and GPO repeats at the

end of the helix have been preserved across all eukaryotes (2). The locations of the sponge collagen cross-links are unknown, although sponge collagens have characteristic D-periods and (modified) lysine in the correct places for cross-linking, so the cross-links are also likely to be conserved.

We also examined collagen intron sequences. The first six bases at the 5' end of many introns are identical or very similar as they are splicing sites, along with the last 3 bases of the 3' end (3), and a graph similar to a phylogenetic tree could be constructed relating the exons. However, moderately detailed analysis of these sequences did not shed any light upon the manner that the exons were duplicated to lengthen the gene.

Comment on Gene Duplication:- Gene duplication was proposed as a potential enabler for evolution of new functions. The concept allows mutation, subfunctionalization, or specialisation of gene copies to occur without lethal effects (4,5). While gene duplication could work in tandem with the proposals here, different collagen chains can also mix to form a hybrid helix, potentially lethal.

Modern cell types avoid this by the intervention of a C-terminal NC1 domain that brings together appropriate chains to initiate folding. The NC1 domains of vertebrate and invertebrate fibrillar collagens all have sequence similarities (6). The equivalent domains in basement membrane type IV collagen, FACIT collagens, nematode cuticular collagens, and spongins (7) are also similar. These two NC1 domain types are therefore likely to have co-evolved with fibrillar and non-fibrillar collagens.

The ancestral fibrillar gene only diverged upon the completion of the main helix with 57+ exons, implying that this parental gene structure evolved quickly, before shorter fibrillar collagen offshoots became essential for survival. Examination of the N-terminal propetide and genes close by in the genome show that fibrillar collagens subsequently separated into 3 clades. Clade B was copied with the NOTCH, complement, and tenascin genes, and includes collagens type Vα1, Vα3, XIα1, XIα2. Clade A was copied with the HOX cluster genes (8), and includes the remaining fibrillar collagen chains of type I-V. Recently, a small C-clade was characterised; it includes the fibrillar collagens XXIV and XXVII (9).

Comment on Exon Shuffling:- DNA analysis suggested that exon groups encoding an entire domain can be duplicated and inserted back into a gene (4,10). Likewise, the more common importing of exons coding for a new domain on one end of the existing protein (4) is similar to some of the steps outlined here. All the exons coding for the COL domain of fibrillar collagens are of the same phase, coding exactly for $(GXX')_n$, allowing this proposal to reinforce work suggesting that exon shuffling can extend a repetitive domain, as well as add new domains (7,11). The lack of similar machinery to copy and insert exons in prokaryotes can explain why they only have a few, much shorter, collagen-like proteins (7).

Comment on Alternative Splicing:- One function of alternative splicing is to prevent lethality should a duplicated exon cause a phase shift. Only one of two possible phase-shifting exons can be spliced in. Another function is for a shorter isoform to be a dominant-negative regulator of the longer form's activity (12). While neither of those functions is suggested here, two exons could be duplicated and back-inserted by a transposon, and then be alternatively spliced in to replace an existing exon. However, alternative splicing rarely involves adding in of exon groups, and is much more commonly used to insert, remove, or replace just one exon (13). This mechanism is therefore likely to enable exon additions rather than being disruptive due to the addition or subtraction of large numbers of exons in one block. Alternative splicing has not been observed within the COL domain, rather it occurs most noticeably in the non-helical domains from collagen types II and XI (14,15).

Comment on Proline 4-Hydroxylase:- The model assumes that there is proline hydroxylase activity, pre-exisiting and co-evolving with the first exon, that specifically modifies the X'-position to 4-hydroxyproline, because the initial $(GPP)_6$ coding exon is otherwise not long enough to code for a stable helix. While this 4-hydroxylation confers a huge stability advantage to the helix and thus enables more functionality by allowing a lower imino acid content of the helix, it is reasonable to argue that it could evolve after the helix is established. As a result, the initial exon coding for $(GPP)_6$ may not have resulted in a stable helix until a the second exon coding for $(GPP)_5$ was spliced in, since $(GPP)_{11}$ is a stable helix in cold water while $(GPP)_6$ is not (16). There is a strong connection between the stability of collagens and the body temperature of the organism (17-19), which would be severely disrupted if all X' position prolines were suddenly 4-hydroxylated, so either the proline 4-hydroxylase(s) evolved early in this process, or its modification of X'

position prolines was increased in a progressive and controlled manner, probably by changes in the enzyme specificity. Additionally, as $(GPP)_n$ proteins without modification are very hydrophobic, hydroxylation and point mutation to amino acids maintains protein solubility and stability, becoming more important as the peptide lengthens

Comment on Hybrid collagens:- Non-lethal hybrid fibrils are possible even if the organism has a mixture of two collagen genes which code for collagens of similar size. We show one hybrid fibril where the gene is at the transition between steps **1 and 2** in figure 2a **(top, suppl. fig. 1)**, where cross-sections A-C are detailed (middle, suppl. fig 1) and a second hybrid fibril between steps **5 and 6 in figure 2c (bottom, suppl. Fig 1)**. These fibrils have a more complex D-period pattern, and ordered packing either as shown in the middle cross-section or with sheet-like cross-linking **(figure 3**). Any hybrid stages would be brief, almost certainly impact the organism, and will require the development of collagenous NC1 domains to ensure the correct chains are folded to form each helix. It is therefore more likely that there was only one evolving gene in the unicellular metazoa ancestor.

Evidence from collagen binding proteins:- **Figure 2c** suggests that the last few additions to the gene are likely to have included multiples of 54-base exons, where the latter half of the gene has long stretches of contiguous 54bp exons. The stretch towards the C-terminal of the molecule but before the C-terminal helical cross-linking site is therefore likely to be the youngest stretch in the collagen. It is void of any major integrin, vwf, or glycoprotein VI binding sites (20), implying that those functions may have been established before collagen reached its final length. This is a reasonable argument given that integrins have been identified in sponges (21) and thus may have co-evolved with collagen.

Calculation of the 1.3% figure for the probability of 23 contiguous 54-base pair exons:- We generated a random sequence of 48 exons; being the number of exons between the two cross-linking sites. 39 were 54-base pair exons, whilst 9 were 45-base pair exons. This randomization was run 240,000 times, and the frequency of a 23 or longer sequence of 54-base pair exons recorded. The probability result given more precisely is 1.254 +/- 0.021%.

Potential proof of Theorem.:- This model is at least partially testable. Collagen genes can be modified to remove exons, and the resultant fibrillar structures analysed. If the properties do not change in the manner predicted by these proposals, this evolutionary pathway would be falsified. We were also encouraged to see that many of the fibronectin and IG domains in titan and fibronectin were coded by one or two exons. This is shown in **suppl. tables 4 and 5**, where the full sequence from these two proteins is split up into domain fragments.

Additional detail on the figures in main paper:-

Figure 1: The exon structures of the type Vα2 and XIα2 collagen genes are identical to their respective α1 chains within the main helix, although they vary elsewhere.

Figure 3: This is shown simplified on three counts for the sake of clarity.

First, modern collagens have been shown to be kinked during the gap region (22). As a result, helices labelled “2” in the “A” cross-sections undergo translation to another “2” position in the “B” cross-section in a modern collagen,

Second, as stated, all collagens have their chains staggered by a single residue so that glycine in any one strand will be adjacent in the helix to X and X' in the other two strands. Each helix therefore has a leading strand (the most N-terminal), a middle strand, and a trailing strand. As a result, the cross-links in figure 3 are not at exactly the same position along the horizontal helical axis.

Third, the three cross-linking lysine residues at each of the N and C-termini of the helix are not offset an exact 120° from each other. The rotational offset between 2 lysines from two adjacent chains (leading-middle, or middle-trailing) is actually between 103° (a 7_2 helix) and 108° (a 10_3 helix). Functionally however, this will still result in the structures as shown in figure 3 or close analogues.

Figure 4(top): An alternative is that the helix 2 group is axially rotated by 120° compared to helix 1. This restores the Gly-X-X’ packing in the overlap region between helix 1 and helix 2 seen in cross-section A. However, this causes any specific binding sites to be rotated around on the surface of the collagen by

120° per D-period where previously they were not. This alternative is also not possible when the collagen has 3 or more D-periods, as X-ray structures of fibrillar collagens show collagen molecules translated in position compared to nearby molecules, rather than translated and rotated.

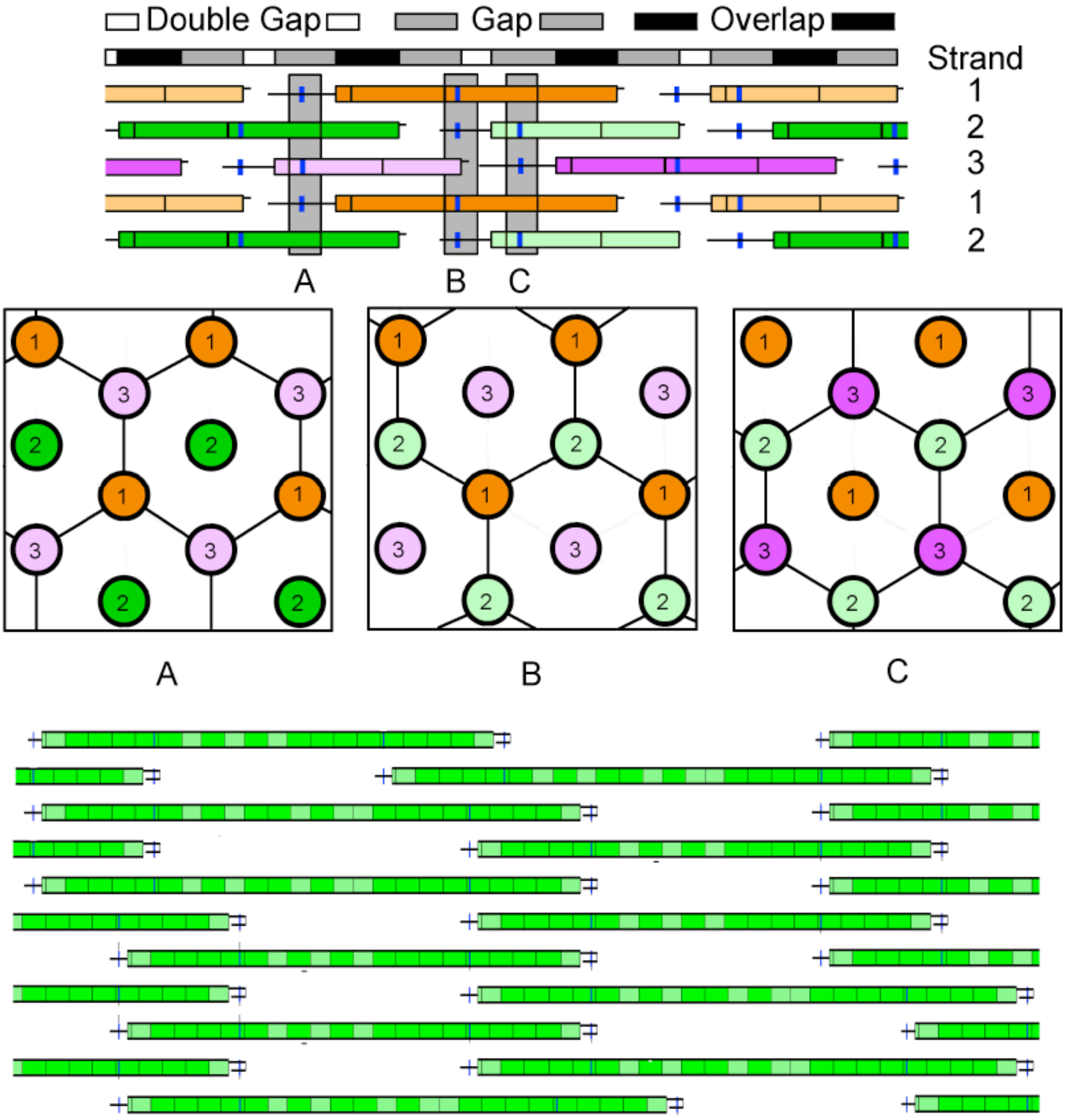

Supplementary figure 1:- *Hybrid collagens.* It is possible to construct fibrillar collagens comprising more than one size of collagen. This is likely to only be a temporary situation before a single collagen takes over, as there would be complications on how the cell builds such a fibre.

SUPPLEMENTARY TABLES

Supplementary table 1. *An evolution sequence to form the ancestral fibrillar collagen.* There are several other possibilities over which exons are added when, provided they are the correct size each time. The evolutionary step as corresponding to figs. 2a-c is shown at the top of each table. Key:
§-see p1, suppl. text;
*-The terminal exons will have telopeptide-coding sequence attached on the N and C-terminal;
R-step is a rearrangement to include an extra D-period;
CL- step is an addition of a second cross-link set; XL-cross-link.
In Step c7of table 1c, either an excision and re-insertion of a 2 exon fragment is required, there needs to be a contiguous four-exon fragment retained as a mobile element, such one from exons 12,13,23,33 which are sequential in step c6.

TABLE 1a - STEP	a1	a2	a3	a4	a5	a6	a7	a8	a9	a10	a11	a12
Possible exons added	N,54(XL),C	6	7	1	2	55	3	56	4	57	5(XL)	XL
Exon lengths	*9,54,45*	54	45	45	54	54	54	54	54	54	54	-
D-periods	4§	3§	2	2	2	2	2	2	2	2	2	2
D-period length§	12	30	45	60	78	78	96	96	114	114	132	132
Gap	12§	36§	21	36	54	36	54	36	54	36	54	54
Overlap	0§	-6§	24	24	24	42	42	60	60	78	78	78
N-term. to XL.	6	24	39	54	72	90	90	108	108	126	126	126
XL to C-term.	31	31	31	31	31	31	49	49	67	67	85	85
total length§	36	54	69	84	102	120	138	156	174	192	210	210

TABLE 1b - STEP	b1	b2	b3	b4	b5	b6	b7	b8	b9	b10	b11	b12
Possible Exons added	N,5(XL),C	6	2	55	3	56	4	57	1	7	54(XL)	XL
Exon lengths	*9,54,45*	54	54	54	54	54	54	54	45	45	54	-
D-periods	2	2	2	2	2	2	2	2	2	2	2	2
D-period length§	27	45	45	63	63	81	81	99	99	114	132	132
Gap§	18	36	18	36	18	36	18	36	21	36	54	54
Overlap§	9	9	27	27	45	45	63	63	78	78	78	78
N-term. to XL.	18	18	36	36	54	54	72	72	87	87	87	87
XL to C-term.	19	37	37	55	55	73	73	91	91	109	124	124
total length§	36	54	72	90	108	126	144	162	177	192	210	210

Table 1c - STEP	ab12	c1	c2	c3	c4	c5	c6	c7*	c8	c9	c10	c11	c12	c13	c14	c15
Exons added	-	8<br>9	10<br>11	12<br>13	23,<br>33	R	29-<br>32	14,15<br>21,22	R	16-<br>20	24-<br>28	34-<br>38	39-<br>43	44-<br>48	49-<br>53	R
Exon lengths (bp)	-	54<br>45	54<br>45	54<br>45	45<br>54	-	45<br>45<br>54<br>54	54<br>45<br>45<br>54	-	54<br>54<br>54<br>54<br>54	54<br>54<br>54<br>54<br>54	54<br>54<br>54<br>54<br>54	54<br>54<br>54<br>54<br>54	54<br>54<br>54<br>54<br>54	54<br>54<br>54<br>54<br>54	-
D-periods	2	2	2	2	2	3	3	3	4	4	4	4	4	4	4	5
D-period length	132	165	198	231	264	132	165	198	132	162	192	222	252	282	312	234
Gap	54	87	120	153	186	54	87	120	54	84	114	144	174	204	234	156
Overlap	78	78	78	78	78	78	78	78	78	78	78	78	78	78	78	78
N-term to XL	87	87	87	87	87	87	87	87	87	87	87	87	87	87	87	87
XL to XL - Residues	39	72	105	138	171	171	237	303	303	393	483	573	663	753	843	843
XL to XL - Turns	1.33	2.33	3.33	4.33	5.33	5.33	7.33	9.33	9.33	12.33	15.33	18.33	21.33	24.33	27.33	27.33
XL to XL-Res/turn	29.3	30.9	31.5	31.8	32.1	32.1	32.3	32.5	32.5	31.9	31.5	31.3	31.1	30.9	30.8	30.8
XL to C-term	85	85	85	85	85	85	85	85	85	85	85	85	85	85	85	85
Full Length	210	243	276	309	342	342	408	474	474	564	654	744	834	924	1014	1014

Supplementary table 2 legend :- *An alternative evolution template should the second set of cross-links have formed at a different point.* This means collagen b12 is different, in this case having a length of 249, D-period length of 113, and overlap length of 96. Key – as Suppl. fig 1. Steps which effectively involve the addition of the same set of exons are concatenated. N/a – not applicable, as previous steps not shown.

Table 2-STEP	b12	c1-5	c6	c7-8	c9	c10-14	c15	c16	c17	c18-19	c20	c21-22	c23	c24-25	c26
Exons added	-	n/a	-	n/a	-	n/a	-	-	-	n/a	-	n/a	-	n/a	-
Exon lengths (bp)	-	45 54 five times	R	45 54 45 54 twice	R	54 x5 five times	R	R	R	54 x10 twice	R	54 x10 45 x2 twice	R	54 x10 45 x4 twice	R
D-periods	2	2	3	3	4	4	5	6	7	7	8	8	9	9	10
D-period length	153	318	159	225	150	300	225	180	150	210	180	240	210	270	240
Gap	57	222	63	129	54	204	129	84	54	114	84	144	114	174	144
Overlap	96	96	96	96	96	96	96	96	96	96	96	96	96	96	96
N-term to XL	105	105	105	105	105	105	105	105	105	105	105	105	105	105	105
XL to XL - Residues	39	204	204	336	336	786	786	786	786	1146	1146	1566	1566	2046	2046
XL to XL – Turns	1.3	6.3	6.3	10.3	10.3	25.3	25.3	25.3	25.3	37.3	37.3	51.3	51.3	67.3	67.3
XL to XL-Res/turn	29.3	32.2	32.2	32.5	32.5	31.0	31.0	31.0	31.0	30.7	30.7	30.6	30.6	30.4	30.4
XL to C-term	107	107	107	107	107	107	107	107	107	107	107	107	107	107	107
Full Length	249	414	414	546	546	996	996	996	996	1356	1356	1776	1776	2256	2256

Supplementary Table 3 legend :- *An alternative evolution pattern for a collagen with* 7_2 *helical symmetry.* Key- as Suppl. tables 1a-c, with concatenation of similar steps as for Suppl. table 2.

Table 3a -STEP	b1	b2	b3	b4	b5	b6	b7	b8	b9	b10	b11
Possible Exons added	*XL(5),C(47)*	46	N(1)	45	2	18	3	44	4	43(XL)	-
Exon lengths	*63,63*	63	63	63	63	63	63	63	63	63	XL
D-periods	2	2	2	2	2	2	2	2	2	2	2
D-period length§	39	60	60	81	81	102	102	123	123	144	144
Gap§	36	57	36	57	36	57	36	57	36	57	57
Overlap§	3	3	24	24	45	45	66	66	87	87	87
N-term. to XL.	12	12	33	33	54	54	75	75	96	96	96
XL to C-term.	31	52	52	73	73	94	94	115	115	136	94
total length§	42	63	84	105	126	147	168	189	210	231	231

Table 3b-STEP	b1	b2	b3	b4	b5	b6	b7	b8	b9	b10	b11
Possible Exons added	*N(1),XL(43)*	2	C(47)	3	46	18	45	4	44	5(XL)	-
Exon lengths	*63,63*	63	63	63	63	63	63	63	63	63	XL
D-periods	2	2	2	2	2	2	2	2	2	2	2
D-period length§	39	60	60	81	81	102	102	123	123	144	144
Gap§	36	57	36	57	36	57	36	57	36	57	57
Overlap§	3	3	24	24	45	45	66	66	87	87	87
N-term. to XL.	33	54	54	75	75	96	96	117	117	138	96
XL to C-term.	10	10	31	31	52	52	73	73	94	94	94
total length§	42	63	84	105	126	147	168	189	210	231	231

Table 3c- STEP	b11	c1-6	c7	c8-10	c11	c12-15	c16	c17-19	c20
Exons added	-	6-11	-	12-17	-	19-30	-	31-42	-
Exon lengths (bp)	-	63 6 times	R	63 x2 3 times	R	63 x3 4 times	R	63 x4 3 times	R
D-periods	2	2	3	3	4	4	5	5	7
D-period length	144	270	135	198	132	216	162	225	150
Gap	57	183	48	111	45	129	75	138	63
Overlap	87	87	87	87	87	87	87	87	87
N-term to XL	96	96	96	96	96	96	96	96	96
XL to XL -Residues	42	168	168	294	294	546	546	798	798
XL to XL –Turns	2	8	8	14	14	26	26	38	38
XL to XL- Res/turn	21	21	21	21	21	21	21	21	21
XL to C-term	94	94	94	94	94	94	94	94	94
Full Length	231	357	357	483	483	735	735	987	987

Supplementary table 4 Legend: *Domain structure of Titin.* Most individual domains are coded within a single exon (**1 exon**). Frequently, exons coding a domain merged to form a larger exon **(merged exons)** that may code for more than one domain. Occasionally, two or more exons are used to code a domain **(2 exons, 3 exons)**. The domain types and zoning of the protein are described in previous work (23) which set out the 363-exon structure of the gene. Depending on alternative splicing and tissue type, different Titin isoforms are made. There are many opportunities for domain duplication to have occurred during the evolution of Titin with functionally analogous effects to adding in collagen sequences to build the collagen gene as described here.

(a) Three of these 2-exon domains are copies of each other, a fourth domain has 2 exons, but one of the exons is only two residues.

(b) Many of the merged domains in the I-Band are derived from the Novex-1 to 3 alternative splicing options for titin, where alternative splicing-in of these domain clusters terminate titin at about 1/5 of its normal size.

(c) The PEVK repeats contain 3 sets of 9 PEVK domains that are copies of each other (23).

(d) The A-band has a 7-domain super-repeat of FnFnIgFnFnFnIg repeated 7 times, and an 11-domain super-repeat of FnFnFnIgFnFnIgFnFnFnIg repeated 9 times.

Titin Zone	Domain type	total	1exon	2exons	3+exons	Merged exons
Z-Line	Ig-like	7	2	5	0	3
	Z-repeat	7	7	0	0	1
	Unique	6	5	0	1	4
Z-I Junction	Ig-like	2	2	0	0	2
	Unique	3	3	0	0	3
I-Band + I-A Junction	Ig-like	107	97	10(a)	0	17 (b)
	Fibronectin-III	11	7	4	0	0
	PEVK (c)	125	125	0	0	18
	Unique	20	18	2	0	15
A-Band (d)	Ig-like	49	48	0	1	26
	Fibronectin-III	121	118	3	0	64
M-line	Kinase	1	1	0	0	1
	Ig-like	10	8	2	0	9
	Unique	8	8	0	0	7
SUM	n/a	477	449	26	2	170

Supplementary table 5 Legend: *Domain Structure of Fibronectin (24).* Fibronectin (Fn) is nearly entirely made up of the 3 types of Fn domain, where the exon lengths coding for any one of the domain types are somewhat variable. Unlike collagen, Fn does not continue key secondary structure elements from one exon to the next, giving it more flexibility. Rather, each exon normally codes for its own separate domain. Under those restrictions, Fn domains may have been copied and reinserted to build up the protein, although this will have necessarily needed to be more complex in the case of the Fn-III domain, which usually is coded by 2 exons.

(a) The exon coding for the 17th Fn-III repeat is usually spliced out, leaving 16 Fn-III domains in a normal Fn protein (25).

Domain type	total	1exon	2exons	Merged exons
Signal Seq	1	1	0	0
Fibronectin-I	12	11	1	1
Fibronectin-II	2	2	0	0
Fibronectin-III (a)	17	5	12	1
Unique	1	1	0	1
SUM	33	20	13	3

Supplementary references.

1. Hofmann, H., Fietzek, P. P., and Kuhn, K. (1980) *J Mol Biol* **141**, 293-314
2. D'Alessio, M., Ramirez, F., Suzuki, H. R., Solursh, M., and Gambino, R. (1989) *Proc Natl Acad Sci U S A* **86**, 9303-9307
3. Yamada, Y., Avvedimento, V. E., Mudryj, M., Ohkubo, H., Vogeli, G., Irani, M., Pastan, I., and de Crombrugghe, B. (1980) *Cell* **22**, 887-892
4. Ekman, D., Bjorklund, A. K., and Elofsson, A. (2007) *J Mol Biol* **372**, 1337-1348
5. Hughes, A. L. (1994) *Proc Biol Sci* **256**, 119-124
6. Boot-Handford, R. P., and Tuckwell, D. S. (2003) *Bioessays* **25**, 142-151
7. Exposito, J. Y., Cluzel, C., Garrone, R., and Lethias, C. (2002) *Anat Rec* **268**, 302-316
8. Sicot, F. X., Exposito, J. Y., Masselot, M., Garrone, R., Deutsch, J., and Gaill, F. (1997) *Eur J Biochem* **246**, 50-58
9. Exposito, J. Y., Valcourt, U., Cluzel, C., and Lethias, C. (2010) *International journal of molecular sciences* **11**, 407-426
10. Liu, M., and Grigoriev, A. (2004) *Trends Genet* **20**, 399-403
11. Patthy, L. (1999) *Gene* **238**, 103-114
12. Kondrashov, F. A., and Koonin, E. V. (2001) *Hum Mol Genet* **10**, 2661-2669
13. Mironov, A. A., Fickett, J. W., and Gelfand, M. S. (1999) *Genome Res* **9**, 1288-1293
14. McAlinden, A., Liang, L., Mukudai, Y., Imamura, T., and Sandell, L. J. (2007) *J Biol Chem* **282**, 24444-24454
15. Medeck, R. J., Sosa, S., Morris, N., and Oxford, J. T. (2003) *Biochem J* **376**, 361-368
16. Persikov, A. V., Ramshaw, J. A., and Brodsky, B. (2005) *J Biol Chem* **280**, 19343-19349
17. Bornstein, P., and Traub, W. (1979) The chemistry and biology of collagen. In: Neurath, H., and Hill, R. L. (eds). *The Proteins*, 3 Ed., Academic Press Inc., New York, NY
18. Leikina, E., Mertts, M. V., Kuznetsova, N., and Leikin, S. (2002) *Proc Natl Acad Sci U S A* **99**, 1314-1318
19. Saito, M., Takenouchi, Y., Kunisaki, N., and Kimura, S. (2001) *Eur J Biochem* **268**, 2817-2827
20. Farndale, R. W., Slatter, D. A., Siljander, P.-M., and E., J. G. (2007) *Journal of Thrombosis and haemostatis, State of the Art series review* **5**,
21. Pancer, Z., Kruse, M., Muller, I., and Muller, W. E. (1997) *Mol Biol Evol* **14**, 391-398
22. Orgel, J. P., Irving, T. C., Miller, A., and Wess, T. J. (2006) *Proc Natl Acad Sci U S A* **103**, 9001-9005
23. Guo, W., Bharmal, S. J., Esbona, K., and Greaser, M. L. (2010) *Journal of biomedicine & biotechnology* **2010**, doi 10.1155/2010/753675
24. Patel, R. S., Odermatt, E., Schwarzbauer, J. E., and Hynes, R. O. (1987) *Embo J* **6**, 2565-2572
25. Schwarzbauer, J. E., Patel, R. S., Fonda, D., and Hynes, R. O. (1987) *Embo J* **6**, 2573-2580
